# On Old Age: A Relational Account of Agency and Meaning in Later Life

**DOI:** 10.1002/hast.5022

**Published:** 2026-02-04

**Authors:** Xavier Symons, Julian Savulescu

**Keywords:** aging, flourishing, later life, old age, end of life, later‐life crisis, bioethics

## Abstract

Cicero's treatise *On Old Age* offers an optimistic account of aging and responds to the prejudiced arguments of those who might otherwise ridicule older members of Roman society. While Cicero's rhetoric is, at times, scientifically naive and moralistic, this article argues that there are important insights that can be gained from carefully theorizing later life as a distinct and valuable stage of human existence—a stage of life that ought not be reduced to a mere proxy for health risk. A careful analysis provides insight into the conditions for flourishing in later life notwithstanding a more pronounced expression of the aging process. Some scholars downplay possibilities for agency and meaning in later life and foreground dignity as an overarching value for old age. We argue, however, that agency and meaning are not only possible but also central in later life but must be supported by social relationships that enhance well‐being and the pursuit and realization of life goals.

## Article

In *On Old Age* (*De Senectute*), the Roman philosopher and statesman Cicero—speaking in a fictional dialogue through Cato the Elder—responds to four misconceptions about aging: that it withdraws us from active pursuits, that it makes one's body weaker, that one cannot experience pleasure, and that death is imminent.^1^ Cicero was right—aging need not imply a grim physical decline, low well‐being, and a withdrawal from life in community. What is missing from Cicero's discussion, however, is an account of how agency and meaning can be preserved in later life. Developing that account is our goal in this paper. Agency and meaning are possible in later life, we will argue, but they are dependent on strong social relationships that enhance well‐being and enable the pursuit of life goals.

Cicero confidently dismisses the four misconceptions, arguing that they all misrepresent the realities of aging, which, in spite of some reduction in capacity, generally still allows one to live a flourishing life. In fact, argues Cicero, the maturity of old age allows for greater intellectual and moral excellence than one is capable of in earlier stages of life. For Cicero, then, the burdens of aging are not at all like what the young Laelius and Scipio, Cato's interlocutors, believe them to be. With reference to the Stoic commitment to a rational principle ordering the cosmos, Cato asserts that, if Nature “has fitly planned other acts of life's drama, it is not likely that she has neglected the final act as if she were a careless playwright.”^2^ Later life has a moral purpose, then, and this purpose allows one to look beyond the biological constraints of age.

Granted, there is a naivety about Cicero's dialogue when read in light of contemporary geriatric science and evolutionary understanding of the “designs” of nature. Cicero dismisses the specter of dementia in old age, even though age is the biggest single risk factor for developing what today are diagnosed as Alzheimer disease and several other dementias.^3^ There is also remarkable variation in the manner in which people age. Many of Cicero's confident ripostes seem to be, at best, sweeping or wishful generalizations or, at worst, problematic ascriptions of responsibility for a loss of physical and cognitive function. Plato may have died “pen in hand, in his eighty‐first year,”^4^ but many people experience chronic illness in later life that leaves them pained and frustrated in their final years and unable to pursue many of their favorite activities or even to use simple tools.

Cicero was a great rhetorician. This paper seeks to preserve something of the spirit of his philosophical dialogue in offering a reflection on flourishing in later life—an account that is cognizant of both the Ciceronian ideal and the complex realities of aging. His central insight—that meaningful, objectively valuable activity and existential engagement in later life are possible—is important for times like our own, in which ageist assumptions can condition our own perspectives on the possibility of flourishing. But he seems to neglect the role of relationships in a flourishing later life. This paper seeks to buttress Cicero's argument by considering how agency and meaning are possible in later life through the support of social relationships that facilitate the pursuit and realization of life goals.

We begin our discussion by offering an account of later life as a distinct life stage that ought not be reduced to a mere proxy for health risk. We discuss the possibility and importance of agency and meaning in later life and criticize an alternative view, advanced by Nancy Jecker, that dignity takes priority over other values in later life. The final section of this paper explores how social relationships provide a scaffold for agency and meaning in later life, and we conclude with reflections on how to preserve the central insight of Cicero's argument.

## Defining Later Life

There are different periods or stages in the course of a typical human life that have unique moral and existential significance. These stages are defined by a range of factors, including raw biological facts about aging as well as a range of social and cultural factors that condition the roles that people occupy. The terminology surrounding life stages and concepts like *life course* and *life cycle* are notoriously ambiguous across the social sciences.^5^ But there is a common commitment to distinguishing stages of human life and identifying the unique biological, economic, social, and cultural factors that characterize these distinct stages.

There is remarkable sociological variation in how societies conceive of what it means to be old.^6^ The United Nations variously defines older persons as those sixty or sixty‐five and older,^7^ while some European Union authorities fix the starting age for this category at sixty‐five.^8^ Discussions of older age are often indexed to retirement age and pension eligibility, the threshold for which in developed countries is somewhere between sixty and sixty‐seven years of age.

We should, however, distinguish different subgroups of people who may be considered old but whose circumstances and life experiences differ dramatically. People who are in their sixties and still active have a very different life experience from people who have entered their eighties and nineties and whose activity may be significantly reduced. Some gerontologists conceive of *old* as a spectral concept ranging from the *young old* (fifty‐five to sixty‐four), through *mid‐old* (sixty‐five to seventy‐four), *old old* (seventy‐five to eighty‐four), and *oldest old* (eighty‐five and above).^9^ Even at the upper echelons of later life, however, we must avoid collapsing *old age* into the *end of life*. The category of end of life is itself quite vague and culturally variable. Deborah Carr and Elizabeth Luth note that “the end of life differs from other life course stages in that scholars and practitioners have not established a consensus definition nor a definitive demarcation of when this stage starts.”^10^ The end of life is characterized by a predominant feature of a close and rapidly advancing time horizon for one's life and the presence of chronic or terminal illness.

Later life, in contrast, can be a time of relative vitality and highly meaningful personal, social, and professional activity. The blurring of later life with a grim end‐of‐life discourse overlooks, for example, the fact that some 19 percent of adults aged sixty‐five and older in the United States are still employed—and mostly employed full time.^11^ The other 80 percent may be vigorously pursuing post‐retirement life projects, caring for grandchildren, or volunteering in the community.

## On the Centrality of Agency and Meaning in Later Life

We should seek, then, to capture the existential meaning of later life and avoid a biological reductionism that can rob later life of its meaning. As Carl Jung wrote, “The afternoon of life is just as full of meaning as the morning; only, its meaning and purpose are different.”^12^ Some of the features of later life may have a positive valence, such as mature and meaningful relationships, opportunities to pursue hobbies, and opportunities for moral and spiritual growth. Some may have a mixed or negative valence, such as health risks, precarious employment or living arrangements, bereavement, or the suffering of significant others who have their own aged‐related health problems. But crucially, the presence of age‐related factors that might condition one's capacities by no means need to constrain agency or one's core interests in life. This is the key mistake in popular discourse on aging. Ezekiel Emanuel for example, wrote in a famous 2014 *Atlantic* article, “Why I Hope to Die at 75,” “[L]iving too long is also a loss. It renders many of us, if not disabled, then faltering and declining, a state that may not be worse than death but is nonetheless deprived. It robs us of our creativity and ability to contribute to work, society, the world.”^13^

Contra Emanuel, later life entails a more pronounced manifestation of aging but—given its clear distinction from life's end—also an open domain of possibility, experience, meaning, and achievement. Many people do not experience functional decline until very late in their lives. Thus, later life is characterized by the risk of illness or some presence of illness but not necessarily a predominance of illness. It may involve a change of pace but does not imply a total loss of pace. It is a dynamic stage for one's identity but does not presuppose an annihilation of identity or the passive acceptance of some predefined script whereby one quietly withdraws from professional work or other social roles.^14^

In lieu of this, the focus ought to lie on the opportunities for meaning and existential engagement that later life presents. There is an ancient debate about the nature of meaning, whether it is local or global, but we will argue that both sorts of meaning are possible in later life.

***Challenges to the idea of agency and meaning in later life.*** Nancy Jecker argues that the focus in later life ought to be on dignity, rather than autonomy, given that one's bodily capacities are slowly fading away at that time of life. In her 2020 book *Ending Midlife Bias: Values for Old Age*, Jecker seeks to identify values attendant on later life and focuses particularly on dignity. She suggests that, in old age, the desire for dignity comes to predominate over a desire for autonomy: “[A]ging shifts the values we hold highest. Autonomy and self‐determination may once have taken center stage, when, as young adults, we had to figure out our path and chart a plan for our lives. While these concerns do not go away, they are no longer focal. Instead, in old age we become more focused on maintaining human dignity because we are more at risk of losing basic human capabilities.”^15^

Jecker defines dignity in terms of “species integrity,” which refers to the ability of individual members of a species to realize capabilities definitive of their species. “[R]especting the dignity of individual members of a species demands respecting their central capabilities as beings of a certain kind,” she asserts.^16^ Jecker's argument is based on Martha Nussbaum's capabilities approach to human development, and Jecker develops a life‐stage‐specific account of human capabilities that yields guidance for what it means to respect the dignity of older members of the community.^17^

One of the tasks of philosophy and policy, according to Jecker, is to resist the attempt to elide the perspective of older members of the community with people in their prime of life. We need to avoid what Jecker calls “midlife bias”—a focus on autonomy alone—in the values we use to inform aging policy. Instead, we ought to consider how dignity can play a more central role in aging policy and research.

Certainly, it is true that dignity is important in later life, and likewise moral growth and contemplation. Yet many people live very healthy and active lives well into later life and therefore may not be primarily concerned with the preservation of dignity.^18^ Older people with disabilities or chronic health issues—notwithstanding any functional limitations they face—may be focused on preserving agency even more than on preserving dignity. A great number of older people in general, regardless of disability or chronic illness, may be concerned with preserving their ability to engage in various life pursuits and achievements and may be leading lives filled with the kinds of projects that make agency a more predominant concern than dignity.

That people are treated with respect and are duly appreciated still matters tremendously—dignity, one might say, is a *sine qua non* for flourishing in later life. But to say that dignity is or ought to be the master value for later life is unjustified. Rather, values differ between people, and it is better to focus on the various sources of meaning people have in later life and the kinds of support they need in terms of agency to realize these sources of meaning. What constitutes dignity and its place in a life is, at least in part, a matter for each individual to decide—that is, an expression of their autonomy.^19^

***A positive account of agency and meaning in later life.*** Given that aging need not involve diminished agency and meaning, agency and meaning in life ought to be seen as central values in later life, just as they are in earlier stages of life. But how should meaning and agency be defined in the later life context?

By “meaning in life,” we have in mind the presence of both subjective engagement and objective value. This is the definition of meaning in life as it is defended by Susan Wolf in her 2010 book *Meaning in Life and Why It Matters*. Wolf argues that “[m]eaning arises from loving objects worthy of love and engaging with them in a positive way.”^20^ She notes that “a person's life can be meaningful only if she cares fairly deeply about some thing or things, only if she is gripped, excited, interested, engaged, or … if she loves something. Even a person who is so engaged, however, will not live a meaningful life if the objects or activities with which she is so occupied are worthless.”^21^

Meaning, then, involves both objective value as well as subjective engagement or attachment. It is not sufficient just to really value something; we must also value the right sorts of things. Thus, Ingmar Persson and one of us (Julian Savulescu) argue that the meaning of life is adding value to your own life or the lives of others.^22^

Derek Parfit approached this issue not in terms of meaning but in terms of well‐being. He described three philosophical traditions and accounts of well‐being—hedonistic (happiness), “desire‐fulfilment,” and objective list theories. Each has some plausibility. Parfit concluded that an adequate account of well‐being must accord weight to all valuable mental states, encompassing both desire‐fulfilment and objectively valuable activity.^23^ It may be best not only to engage in activities that possess objective value but also to want to engage in such activities, and to derive pleasure from them.

By “agency,” we have in mind the capability that one has, based on internal and external factors, to realize meaning in life. Agency, on this account, is being able to realize that which has objective value and is subjectively engaging. Thus, we might say that an older person who values lifelong education has agency to realize this source of meaning only if they are still lucid and have a university nearby with free courses for students in older age, or at least for those who cannot afford them. Someone who is suffering from cognitive decline or who lives in an isolated location or cannot afford education does not have agency to realize this potential source of meaning in their life.

To reiterate, agency and meaning (or well‐being) are fundamental values for later life. Societies should seek to promote them and use them as a basis for social policy. Agency and meaning are important both for those who are still very active as well as for those who are experiencing functional decline. The latter appear to be the main object of Jecker's argument, but meaning takes on a special importance when functional limitations are more pronounced, as they tend to be in later life. Later life involves a dynamic process of coming to terms with the challenges that characterize aging as a matter of biological and social fact but also hopefully pursuing new ways of engaging the world and shaping one's life, notwithstanding the limitations attendant on one's condition.

There is, of course, a sense in which meaning and agency are important to all older members of the community. Meaning—which we have defined as objective value plus subjective engagement—derives from the set of activities that give deeper purpose and engagement to one's life, whereas agency—which we have defined as internal freedom plus external social support—is a means by which one realizes meaning. These values are the building blocks of a vigorous and active later life.

The possibilities for local meaning and existential engagement in later life are numerous and diverse, often shaped by individual interests, circumstances, and societal influences. Lifelong learning, hobbies and interests, community involvement, volunteering, mentorship, artistic pursuits, travel, and adventure are all possible sources of meaning as one transitions into retirement. It may also be the case that someone has been engaged in lifelong family caregiving rather than paid work. In this case, such a person may want to use their final decades of life to pursue hobbies and interests or indeed continue, albeit in a different way, their role as a family caregiver (such as by transitioning from parent to grandparent).

An important question arises here: is an endless life of leisure sufficient for most older people, regardless of how much they enjoy gardening, travel, or golf? Does such a life possess objective value? One option is to say that these activities are just not sufficient to create even local, personal meaning in someone's life, yet alone provide a broader, cosmic source of meaning for one's life. It would seem that one also needs a sense that the kinds of pursuits in which one is engaged have objective value—that they are recognized as meaningful and significant by others in the world—in addition to whatever subjective pleasure one derives from them. Endless games of golf, infinite opportunities for home gardening, or a round‐the‐world business‐class travel pass may just not be enough for objective value. The issue is with the trivial nature of the activity itself, not its duration or intensity.

But this is a dismissive position, and in fact it is more accurate to say that some fail to find meaning in these things, while others find them very meaningful. Golf can be a social activity that sustains people through older age, likewise with travel to places that have deep personal significance or gardening with other members of the community. These can be more serious and meaningful activities than one might initially think.

Jecker is, however, mistaken to think that dignity necessarily takes priority over agency and meaning when older people lose functional capacity. We think there is opportunity for meaningful moral growth and transcendence in later life even when functional capacity is diminished. The idea that dignity necessarily becomes the master concept in these cases caricatures the experience of those who live their final years with significant physical and psychological limitations and who yet experience enhanced agency and meaning. Processes of moral growth, ego integration, and self‐transcendence in the context of advanced aging may represent an augmentation of a deeper form of agency pertaining to self‐actualization.

In an eloquent essay entitled “On the Question of Aging,” John Cottingham explores Aristotelian and Judeo‐Christian perspectives on aging and considers how old age and the end of life present unique opportunities for moral growth and ego integration for moral agents, not in spite of but precisely because of the realities of senescence and illness in later life. In a gloss on the Aristotelian notion of *entelechy* (the actualization of potential), Cottingham argues that integrity is a structural feature of an organism's flourishing. That is to say that one aspect of an organism's flourishing is the attainment of a kind of organic unity within the function of that organism. With this in mind, he turns to the topic of aging: “Integrity, as its etymology suggests, has to do with wholeness—it involves the fullest integration of the whole self. And given this, the process of moving towards old age emerges as a vital part of the fullest human flourishing: it is precisely that process which (though of course it does not guarantee it) allows space for there to be a sense of meaning and connection over the entire span of a life.”^24^

But isn’t aging slow disintegration of the function of the organism? Cottingham responds that “moral growth [is] a continuous learning process that is lifelong” and that “age and its trials can afford scope for the deepening of that process.”^25^ The wisdom of old age, he contends, allows us to integrate our own life narrative in a way that we might not be capable of in earlier life.

Cottingham's emphasis on moral and spiritual integration overlaps with Jecker's definition of dignity as species integrity; both authors see organismal integrity as an important value in later life. But Cottingham relies on a more explicitly spiritual anthropology and speculates that this process of moral and existential integration is partly contingent on some conception of self‐transcendence that is the culmination of our moral striving in this life.

Cottingham's reflections thus intersect with the paradigm of *gerotranscendence* in gerontology, proposed and expounded by sociologist Lars Tornstam.^26^ This theory describes an existential and spiritual change of consciousness that human beings undergo in later life. The process of gerotranscendence is defined as “a shift in metaperspective from a materialistic and pragmatic worldview to a more cosmic and transcendent one.”^27^ Gerotranscendent individuals may appear to be more withdrawn from the world but in fact experience a deeper existential connection with the universe and exhibit less self‐centeredness and more universal benevolence.

A principal aim of the theory of gerotranscendence is to offer a more positive appraisal of the apparent social withdrawal that many people undergo in later life. Tornstam encourages practitioners and the broader community to consider how becoming more reserved and removed from social life might not necessarily be a sign of mental illness or deterioration. In fact, it need not even have a negative valence. A person going through this process may, instead, be entering a new contemplative stage of their existence that is worthy of respect and reverence. (Of course, they may instead be suffering from loneliness or dementia.)

Again, it is important not to caricature later life in a way that erases much of the experience of a period, beginning around sixty, that may entail decades of vigorous and active life. It seems as though all these analyses, while they may be insightful for people who are “chronically and progressively ill” or have lost “important physical and mental functioning,”^28^ run afoul of the same conflation that appears in definitions of later life, whereby aging is characterized as a rapid and marked loss of function and a decrease of one's engagement in life projects. Gerotranscendence, for its part, is a useful paradigm for appreciating the spiritual character of many people's experience of later life, in particular of those who experience functional limitations.

In sum, meaning is still possible for older people who have significant functional limitations or who are suffering from serious illness. The agency of these people is conditioned by their life circumstances, but it would be a mistake to think that agency is severely diminished or totally absent. Rather, processes of moral growth, ego integration, and self‐transcendence may represent an augmentation of a deeper form of agency pertaining to self‐actualization. We may not be able to play golf or travel or garden when we are old and sick, but we still have the capacity for moral and spiritual maturation.

These considerations notwithstanding, societies and individuals should be careful not to surreptitiously use gerotranscendence as an excuse for not addressing the social isolation and loneliness afflicting older people.^29^ If an older person becomes withdrawn, clinicians should consider whether this is reflective of the emergence of a positive, more contemplative approach to life or whether in fact a person's withdrawal from social interaction is a sign of depression, loneliness, or social isolation. If the latter, we should consider how meaning and purpose are fundamentally a form of shared agency and how we share and realize purposes or life goals together. Given that older members of the community are vulnerable to social isolation, loneliness, and general loss of purpose, societies need to create supports that allow them to thrive in this life stage. Social relationships are crucial to that thriving.

## Social Relationships as a Scaffold for Agency and Meaning

The very nature of aging and later life—where health risks are, for example, more common—makes community conditions especially important for maintenance of meaning, purpose, and vital engagement. In other words, when someone has significant health problems, their potential for flourishing in later life is particularly dependent on their social condition and relationships. In later life, people can still do things, but they also need more help. Loneliness is also a terrible event in older life that we must be aware of and seek social solutions for. Thus, social relationships are needed to address vulnerability and to provide possibility. They also give a person the ability to create the two sources of meaning in life: first, by adding value to their own life (we need help to execute self‐interested desires) and, second, by providing potential to add value to the lives of others—those who support the person or who can be benefited by them.^30^

There are also various other social barriers that may make it harder for older people to pursue meaningful life projects. Older members of the community may, for example, encounter ageist bias in their attempts to apply for work or volunteering opportunities or find that their competence is underestimated when they are working or volunteering. Australian data, for example, suggests that nearly 58 percent of job seekers fifty and over reported discrimination based on age, and 27 percent of those in this age range who were currently employed reported experiencing age discrimination in the past two years.^31^

It is challenging, then, for older members of the community to maintain and actively pursue life opportunities. But this challenge can be addressed with the assistance of others. Virtue in later life may lie in the pursuit of options rather than acceptance of the fading of options. (The latter disposition remains a potential virtue in midlife, though, given that there may be a propensity in that stage to keep all options open or to endlessly try new options to the extent that it negatively impacts wellbeing.) This requires hopeful and at times creative grappling with one's own embodiment in a manner conducive to growth, notwithstanding the vulnerabilities of later life. Part of this is a mindset challenge—it concerns how one chooses to look at one's life and interpret one's circumstances. But it is also a social challenge. One way to address ageism in contemporary societies is to cease to define later life in terms of its polarities—the end of midlife and the end of life—and to recognize the experience that is characteristic of this distinct life stage. Philosophy, for its part, can legitimate this perspective in its discourse on values and life stages by not creating a dichotomy between autonomy on the one hand and vulnerability or dignity in the face of vulnerability on the other.

As argued above, we must resist the impulse to elide the priorities and values held by many in old age by focusing solely on those typical of people in midlife as well as the impulse to define old age as a state of chronic illness or significant functional impairment. Agency seems to come to the fore as a vital value to promote in later life—the ability to do and be the sorts of things that one wants to do and be in this transitional stage, notwithstanding a more pronounced expression of the aging process. But we need a relational approach to agency in the midst of the aging process, and this requires creating new social networks that give the opportunities for flourishing and that support and buttress individual agency at a time of vulnerability and decline. Meaning matters, but the attainment of meaning in someone's life is not just a question of passion. A “later‐life crisis” is defined by a sense in which both subjective engagement and objective value are missing from one's life.^32^ Given this, we ought to consider the importance of socially buttressed meaning: socially supported pursuit of individual projects and of experiences that add value to the lives of others, such as one's grandchildren or community. Like agency, meaning has a social dimension. Communities can provide their older members with both security and opportunities—security so that the structures of their life are not radically threatened and opportunities so that older people can actively grapple with challenges arising in their lives in such a way that they find new sources of meaning and engagement. These things are important, but they might come from family and friends—one is typically more reliant on others in later life.

Illness may play a part in making someone vulnerable in later life, but several other factors can come into play, including retirement, bereavement, divorce, and moving into a new home. This sense of vulnerability can lead to the feeling that the integrity of one's identity and one's fundamental engagement in life are threatened. Oliver Robinson and Alexander Stell characterize a later‐life crisis as a cumulation of stresses in several or all of six domains of one's life—illness in self, illness in a significant other, bereavement, retirement, divorce, and moving house—culminating in despair and a loss of ego integrity.^33^ In a harrowing article, they catalog the themes of fifty‐seven semistructured interviews with sexagenarians describing their experiences of a life crisis in their seventh decade. Some key themes that emerge from the interviews include loss of identity, social disengagement, depression and anxiety, and thoughts of mortality in light of life's reduced time horizon. Perhaps the most common feature was a sense of purposelessness when retiring from full‐time work. One respondent noted that “there are things you lose” when retiring from full‐time work, “and what you do lose are your routines, your friends, those sort of things. And that's not good.’’^34^ Another interviewee noted, ‘‘It was a strange time for me because I’ve always been busy, I’ve always had a project on the go, and now I was able to sort of sit back, go down to the allotment, enjoy doing a bit of weeding.’’^35^

Older people can mitigate the effects of later‐life crises through a free and deliberate lessening of productive activity on one's own terms and the discovery of new social roles specific to retirement that replace one's professional identity. In contrast, factors such as being removed from productive roles and feeling isolation and regret can exacerbate a later‐life crisis. Thus, social roles are important to the levels of stress and sense of threatened identity that one might experience as one gets older. Whereas the challenge in midlife is to be comfortable with the closing of doors, the challenge in later life is not to despairingly assume that all doors are closed. It is also to create the right kind of social network. Robinson and Stell note that crises early in life are defined by the “challenges of becoming embedded in adult roles that come to feel ensnaring and engulfing, while later‐life crises are defined by the challenges and emotional difficulties of losing those roles.”^36^

It is clear, then, that agency concerning when and how one retires, secure and flexible housing arrangements for older members of the community, sufficient pension allowances, and funding for clubs, societies, and houses of worship that have sizable older memberships are all important features of a decent society that enables the flourishing of its older members.^37^ In addition, close social relationships, such as with siblings, children, grandchildren, and close friends, can buttress meaning and agency. The main point is that, as one gets older, one can still have agency and meaning, as well as dignity, but one is more reliant on others to buttress and structure one's opportunities. Aging can be a time for flourishing or high well‐being, but that presupposes social connection. One can rage against the world when one is young, but it is futile and absurd if one is old. Social policy informed by a relational agential account of aging and meaning in later life can help keep agency and meaning possible.

## Toward a Nuanced Understanding of Later Life

Cicero presents an optimistic picture of old age in response to those who sought to caricature and deride it. This article has considered whether there was something that we might salvage from Cicero's portrait, even if we set aside his naiver claims about what's possible in later life. There are ineliminable declines in function brought on by aging. But it is not the case that they entail an indecorous and precipitous decline or a steady disengagement from society. Rather, later life, in the forms that it takes in many instances, is often characterized by precisely the vigor and subjective fulfillment that Cicero suggests in his dialogue. Therefore, we ought not dismiss his reflections as fundamentally outmoded or naive.

Philosophy, like the social sciences, must develop a more nuanced understanding of the perspective of later life. Given the global reality of an aging population, this task is of great importance.

## Acknowledgments

This research project is supported by the National University of Singapore under the NUS Start‐Up grant NUHSRO/2022/078/Startup/13 and by the Singapore Ministry of Health's National Medical Research Council under its Enablers and Infrastructure Support for Clinical Trials‐Related Activities Funding Initiative (NMRC project no. MOH‐000951).
